# An objective approach to model reduction: Application to the Sirius wheat model^☆^

**DOI:** 10.1016/j.agrformet.2014.01.010

**Published:** 2014-06-01

**Authors:** N.M.J. Crout, J. Craigon, G.M. Cox, Y. Jao, D. Tarsitano, A.T.A. Wood, M. Semenov

**Affiliations:** a School of Biosciences, University of Nottingham, Loughborough, LE12 5RD, UK; b School of Mathematical Science, University of Nottingham, Nottingham NG7 2RD, UK; c Computational and Systems Biology Department, Rothamsted Research, Harpenden, Herts AL5 2JQ, UK

**Keywords:** Crop model, Wheat, Evaluation, Complexity, Model reduction, Parsimony

## Abstract

An existing simulation model of wheat growth and development, Sirius, was evaluated through a systematic model reduction procedure. The model was automatically manipulated under software control to replace variables within the model structure with constants, individually and in combination. Predictions of the resultant models were compared to growth analysis observations of total biomass, grain yield, and canopy leaf area derived from 9 trials conducted in the UK and New Zealand under optimal, nitrogen limiting and drought conditions. Model performance in predicting these observations was compared in order to evaluate whether individual model variables contributed positively to the overall prediction. Of the 1 1 1 model variables considered 16 were identified as potentially redundant. Areas of the model where there was evidence of redundancy were: (a) translocation of biomass carbon to grain; (b) nitrogen physiology; (c) adjustment of air temperature for various modelled processes; (d) allowance for diurnal variation in temperature; (e) vernalisation (f) soil nitrogen mineralisation (g) soil surface evaporation. It is not suggested that these are not important processes in real crops, rather, that their representation in the model cannot be justified in the context of the analysis. The approach described is analogous to a detailed model inter-comparison although it would be better described as a model intra-comparison as it is based on the comparison of many simplified forms of the same model. The approach provides automation to increase the efficiency of the evaluation and a systematic means of increasing the rigour of the evaluation.

## Highlights

• We demonstrate the application of systematic model reduction to a complex crop model.
• The model was manipulated under software control replacing variables with constants.
• Redundancy in representation of nitrogen physiology and temperature was found.
• The level of detail in Sirius is defensible if detailed prediction is required.
• The approach increases the efficiency and rigour of model evaluation.

## 1 Introduction

Simulation models that predict the yield of agricultural crops from weather, soil and management data have provided a focus for crop physiological research over the last three decades and have contributed to current understanding of crop-environment interactions. Many such models have been developed for a wide range of crops, for example, STICS (Brisson et al., 2003), APSIM (Keating et al., 2003), and DSSAT (Jones et al., 2003). The growth and development of agricultural crops in the field is the result of non-linear and inter-related processes, and as a result crop models are necessarily complex. Even when a model approximates individual processes by relatively simple relationships, there are a large number of inter-acting processes that have to be considered. Therefore the complexity of crop models typically arises from the inter-relationships between modelled mechanisms rather than the sophistication of individual process representation. The level of detail, the number of processes considered, and the means whereby they interact are all choices to be made in the design of the model leading to a very diverse range of crop model designs and, as a result, a need for effective methods of model evaluation. The purpose of a model is vital in defining the approach taken to its design and evaluation (Jakeman et al., 2006). For Jamieson et al. (1998b) the explicit aim of a crop model was improved understanding of the crop's response to its environment. They described a crop model as a ‘…collection of testable hypotheses…’ and viewed model inter-comparison as a method of testing the hypotheses embedded in the models through an examination of their ability to predict detailed within season measurements of the crops growth and development. For example Jamieson et al. (1998b) presented a comparison of the performance of 5 wheat models with respect to crop and environmental data from within the growing season. Their conclusions were directed towards the mechanistic basis of the different models. For example the assumptions about the role of root distribution on water uptake and the influence of water stress on canopy expansion were highlighted as important differences in the models considered.

Many researchers (Crout et al., 2009; Cox et al., 2006; Anderson, 2005; Kimmins et al., 2008; Bernhardt, 2008; Smith et al., 2010) have emphasised the need for a systematic evaluation of model structure. Crout et al. (2009) proposed a conceptually simple method for undertaking an evaluation of model structure by reducing a model through the replacement of variable quantities with constants. By iteratively replacing different variables in combination with one another a set of alternative model formulations were created. The ability of the reduced models to predict observations was then compared with the original model in order to test the importance of the replaced variables in the model. To date published work with this approach has considered relatively simple models (e.g. Tarsitano et al., 2011). In this work we extend the approach to the more challenging case of a full crop simulation model with the aim of explicitly testing the hypotheses of the model. The usefulness of the analysis is dependent on the reliability and comprehensiveness of the observational data used. Inevitably the data available are partial, and therefore any model analysis is limited to some extent. Nevertheless we argue that this approach provides greater support for the model design than simply comparing the predictions of the full model with observations.

## 2 Methods

### 2.1 Sirius model

A typical example of a process-based wheat model is Sirius. This model calculates biomass production from intercepted photosynthetically active radiation and grain growth from simple partitioning rules (Jamieson et al., 1998b) utilising nitrogen response and phenological development sub-models described by Jamieson and Semenov (2000). Sirius is an actively developing model currently being applied at a number of levels, from basic research to on-farm decision support (e.g. Semenov and Shewry, 2011; Semenov et al., 2009).

### 2.2 Observational data and model calibration

The reduction approach requires a quantitative comparison between model predictions and observations. In principle this can be based on any relevant data series available. Our purpose was to mechanistically evaluate the performance of the model in reproducing the pattern of growth and development within a growing season as well as between sites and seasons (i.e. testing the description of a growing crop not just the final yield). We therefore selected data from trials where detailed growth analysis had been conducted including cases where the major abiotic stresses of nitrogen and water limitation were present.

Data from 9 trials have been used for the analysis (Table 1): (i) a spring wheat study at Lincoln New Zealand with four levels of water supply (ii) winter wheat trials at three sites in the UK with high nitrogen application rates and (iii) a further UK winter wheat trial with two levels of nitrogen application.

Typically crop models are calibrated for use with particular cultivars and require site specific inputs for weather and soil conditions. Sirius had been previously applied to the New Zealand field data by Jamieson and Semenov (2000) and their cultivar and site parameters were employed for this work. The UK field trials used the cultivar Mercia, for which Sirius had been calibrated previously using field experiments in the UK (Wolf et al., 1996; Ewert et al., 2002; Lawless et al., 2005). Soil and weather characteristics used were as reported by Gillett et al. (1999).

### 2.3 Overview of reduction procedure

The approach was to compare the predictive performance (skill) of a large number of alternative model formulations. These were based on the original full model but with specific variables replaced by constant values. In this context model variables were defined as internal quantities calculated using an assumed relationship expressed in terms of the model's parameters, input variables and other model variables. This definition of model variables was partially subjective because intermediate steps in a model calculation could be defined as individual model variables, or combined into a larger relationship as a single model variable. Such choices often depend upon the requirements of specific computer implementation. However, we regarded each model variable as having a specific mechanistic role in the model and defined a variable as a specific model component whose value was allowed to change during the run of the model (Cox et al., 2006). We therefore tested the effect of fixing a model variable to a constant on the model's skill. If the variable was important for model prediction one would expect replacing it with a constant to have a detrimental effect on the comparison between observations and predictions.

As the assessment of model skill was based on a comparison of observations and model predictions (described below), the approach was explicitly reliant on observational data. The utility of the analysis was therefore directly dependent on the quality and scope of the data used.

The key steps in the analysis were:

1. Implementation. The analysis was undertaken using a software environment which manages the variable replacement on the fly under programmatic control (OpenModel; www.nottingham.ac.uk/environmental-modelling.htm). Therefore the first step was to implement the model within this environment and check its behaviour to ensure it is the same as the original source model. This was accomplished through comparisons to the original hard coded Sirius model.
2. Evaluation of model structure to enable the identification of candidate variables for reduction analysis. Not all the variables in the model merited investigation. For example, many variables in the model code were for diagnostic or output purposes and not part of the models functional structure. Use of the reduction software facilitated a syntactical analysis of the model structure to identify variables which had a technical or operational function in the model code. The remaining 1 1 1 variables, representing the modelled processes, were considered in the screening analysis.
3. Screening analysis. The computational requirements of the reduction procedure increase with the number of variables considered. Therefore a screening analysis was conducted to assist in refining the list of candidate variables. This involved replacing variables individually (one at a time) rather than in combination.
4. Multi-factorial reduction analysis. In this stage the candidate model variables were replaced in combination in order to explore the set of possible model replacements as fully as possible.

### 2.4 Estimation of model performance

Weighted residual sums of squares, RSS, were calculated for each model permutation considered. The weights used were the estimated standard errors of the observations. These were taken as 10, 10 and 20% of the observed value for the biomass, grain weight and LAI values respectively. These fractional standard error values were selected on the basis of experience with crop analysis data sets. Overall model performance was summarised by calculating coefficients of determination (also known as Nash–Sutcliffe model efficiency (Nash and Sutcliffe, 1970)), NS,

(1) $$\text{NS}=1-\frac{{\sum \left(\frac{\left(O_{j}-M_{j}\right)}{s_{j}}\right)}^{2}}{{\sum \left(\frac{\left(O_{j}-\bar{O}\right)}{s_{j}}\right)}^{2}}$$

where *O*_*j*_ and *M*_*j*_ were the *j*th observation and model prediction respectively, *s*_*j*_ is the estimated standard error of the *j*th observation, and $\bar{O}$ was the mean of the observations. NS is analogous to *r*^2^ in regression analysis, the method of calculation is identical, however, the *M*_*i*_ values are for the model considered, not necessarily the best fitting model. Therefore, unlike *r*^2^, NS can be negative.

In previous work (Crout et al., 2009; Tarsitano et al., 2011) we used estimates of the likelihood integrated over the models parameter space (Kass and Rafferty, 1995) as measures of the prediction skill of each reduced model combination. However, in the case of Sirius, model parameters and replacement constants were not fitted for each reduced model combination. Therefore the use of integrated model likelihood was not appropriate and we employed an informal approach relating belief in the model to the weighted residual sums of the squares. This is similar to the use of informal likelihoods as discussed by Beven (2009). A pseudo-likelihood (*Q*) was calculated for each reduced model combination using an informal relationship between the residual sums of the squares of the reduced and full models

(2) $$Q_{i}=A\exp\left(\frac{-\ln(0.5)\left({\text{RSS}}_{\text{full}}-{\text{RSS}}_{i}\right)}{\alpha }\right)$$

where *A* is a normalisation constant such that ∑*Q*_*i*_ = 1 over the models considered; RSS_full_ and RSS_*i*_ are the weighted residual sums of squares for the full model and *i*th reduced model respectively and *α* is a constant whose value controls how changes in RSS affect belief in the model (as measured by *Q*_*i*_). For example, if RSS_*i*_ exceeds RSS_full_ by *α*, *Q*_*i*_ will be half the value of *Q*_full_.

The use of the informal *Q*_*i*_ values as pseudo-likelihoods required cautious interpretation. However our aim was to assess the mechanistic basis of the model, not obtain absolute probability values. The effect of changing the value of *α* on the interpretation was investigated by calculating the *Q*_*i*_*s* with *α* set at 2.5, 5.0 and 10% of the value of RSS_full_.

### 2.5 Screening analysis

In the screening analysis each variable was replaced individually. The value of the replacement constant was estimated by minimising the model residual sum of squares, subject to the constraint that the value must be within the range the variable takes in the run of the full (unreduced) model. These replacement values were used throughout the subsequent analysis.

Variables whose replacement had little or no detrimental effect on model performance were considered for inclusion in the multi-factorial analysis. Where a number of the potential variables related to the same process, switch variables were introduced to the code which enabled the effect of replacing the result of whole process by a constant to be considered. For example, the adjustment of soil maximum temperature from observed maximum air temperature was modelled using a relationship involving two model variables (ENAV and TADJ) calculated elsewhere in the model. Rather than replace these individually it was more convenient to modify the model code and introduce a switch variable to reduce the modelled value of soil maximum temperature to the maximum air temperature, thereby testing the role of ENAV and TADJ simultaneously. Switch variables of this type were introduced for soil minimum temperature, soil maximum temperature and adjustment of canopy temperature from air temperature. In each case replacing the switch variable with zero reduced the temperature variable to the appropriate air temperature. Further switch variables were introduced to reduce the diurnal variation in temperature used for many temperature dependent processes in the model to a daily mean temperature and to switch off the vernalisation sub-model.

### 2.6 Multi-factorial analysis

There are 2^*N*^−1 different possible combinations of replacements for *N* candidate variables. Searching this replacement space is not possible exhaustively for even moderate values of *N*. Therefore a stochastic search based on the Metropolis–Hasting principle was used (e.g. Van Oijen et al., 2005; Hastings, 1970). The state of each candidate variable was either normal or replaced. The procedure moved through the search space by changing these states. For each iteration a step was made to a new model by changing a given number of the variable states (either from normal to replaced or vice versa). The number of states to change is adjusted operationally to achieve a random walk through the search space rather than just a random sample. In the case of Sirius, we found that allowing just one state to change per iteration gave the most efficient search. The results of the analysis were very similar when up to two or three state changes per iteration were allowed but the procedure required a larger number of iterations to converge.

At each step the model predictions were compared to the observed values to calculate the pseudo-likelihood (*Q*; Eq. (2)). The step was accepted if

(3) $$\frac{Q_{\text{trial}}}{Q_{\text{current}}}>r$$

where and where *Q*_current_ and *Q*_trial_ were the pseudo-likelihoods of the currently accepted and trial reduced model combinations respectively and *r* was a random value between 0 and 1.

This Metropolis–Hastings random walk through the replacement space has the ability to accept moves which reduce the model likelihood allowing the walk to escape local minima in the search space. The probability of accepting a bad move is the ratio on the left hand side of Eq. (3) and for each iteration this was compared to the random draw *r*. The effect is that moves with a small detrimental impact on the model fit will be accepted quite often, whereas moves which seriously worsen the fit are unlikely to be accepted. Although the walk through the search space may return to a previously evaluated model this does not adversely affect the search efficiency in our case as the previously calculated pseudo-likelihood value was used avoiding the need to re-evaluate the model.

The pseudo-likelihoods were normalised to unity over the models considered. A replacement probability was calculated for each individual variable by summing the normalised pseudo-likelihoods for the models where the given variable was replaced. The results presented were based on 10,000 unique model evaluations. The replacement probabilities were reported at suitable intervals to ensure they had stabilised after this number of iterations (by simple inspection). The replacement constants used were those obtained for each of the candidate variables in the screening analysis.

## 3 Results and discussion

### 3.1 Full model

**Fig. 1:**
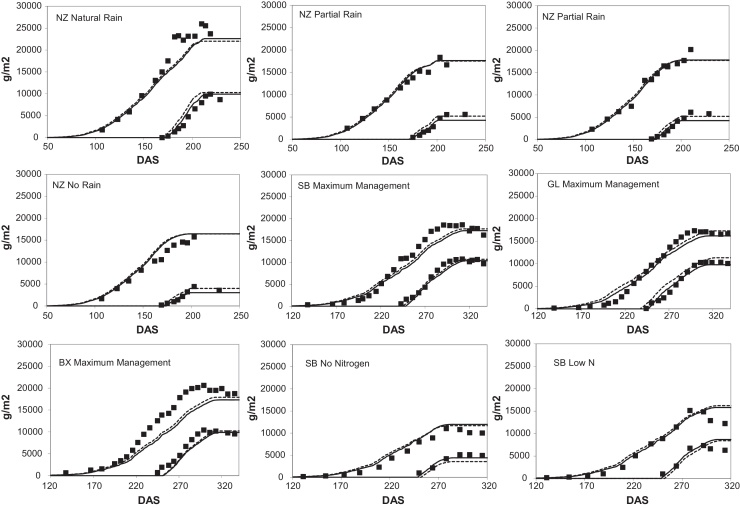
Biomass and grain weight model predictions compared to observations. In all cases grain weight values are in the bottom right of the graph. The full model is the dashed line, reduced model is the solid line.

**Fig. 2:**
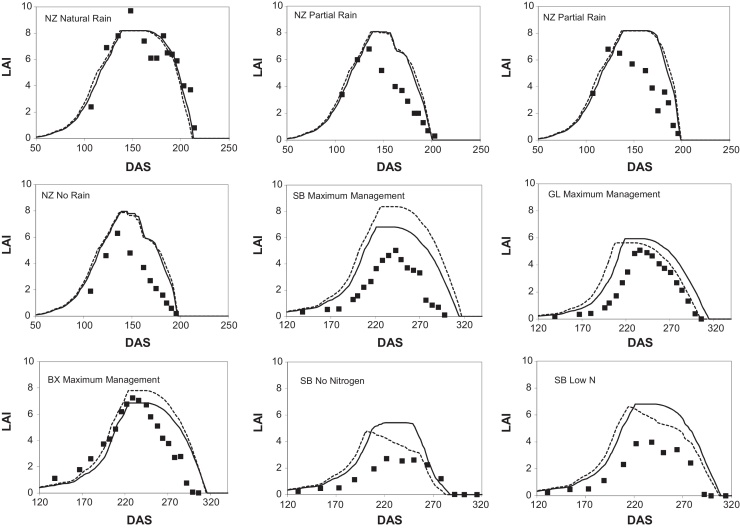
Model predicted canopy leaf area index (leaf area per unit ground area) compared to observations. The full model is the dashed line, reduced model is the solid line.

Predicted and observed total biomass, grain weight and leaf area were compared for the full model (Figs. 1 and 2 respectively) and summarised as Nash–Sutcliffe statistics (Table 2). The trends in biomass and grain weight were well reproduced, although leaf area less satisfactory. The overall timing of the canopy was well described in the model simulations although the canopy size was often over-predicted. In practice over prediction of leaf area tends to be disconnected from the prediction of biomass and grain as light interception does not increase linearly with canopy size. For example, over-prediction of leaf area index from four to five increases fractional interception by only 5% and therefore has little effect on predicted crop production. Under-prediction of leaf area would be expected to have detrimental effects on biomass and grain yield prediction.

### 3.2 Screening analysis

**Fig. 3:**
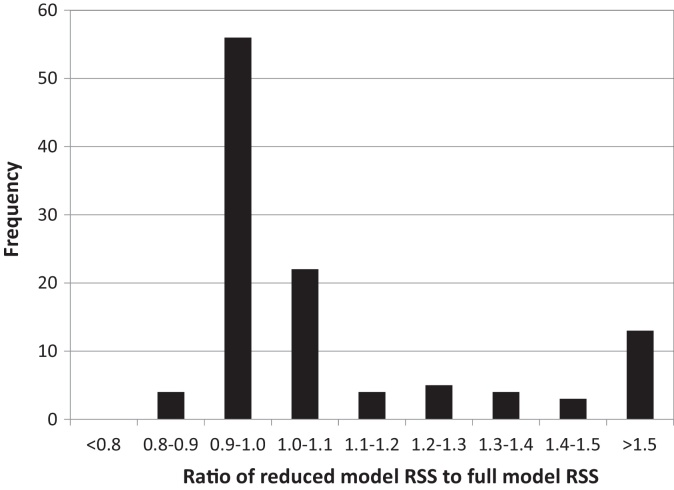
Frequency of the ratio of reduced model RSS to full model RSS for the 1 1 1 variables considered in the screening analysis.

The behaviour of the reduced models in the screening analysis was summarised using the ratio of RSS for the reduced model to that of the full model. The distribution of these values for the 1 1 1 variables considered is shown in Fig. 3. The individual replacement of 29 of the considered variables by a constant increased the ratio of RSS for the reduced model to that of the full model to greater than >1.1. Of the remaining 82 replacements 60 resulted in a smaller RSS than the original full model; the remaining 22 had a small detrimental effect (<10% increase). These 82 variables were considered as potential candidates in the factorial analysis. The reduction of RSS in this screening analysis was expected as the values of the replacement constant for each variable were selected by fitting them to the observed data. However this did suggested that these variables may not be important for the predictive performance of the model.

Up to this point the analysis was entirely automatic. However at this stage mechanistic interpretation of each of the 82 potential variables role in the model was required to ensure that the replacement of specific variables was meaningful. For example some of the variables are intermediate steps in a calculation whose reduction would be best accomplished through the replacement of the final end point variable. The replacement of some variables would break the mass balance of the model, for example allowing it to create nitrogen or dry matter to translocate to the grain irrespective of the status of the crop. On this basis 54 variables were eliminated from the analysis. The remaining 28 model variables, together with 5 switch variables were identified for inclusion in the multi-factorial analysis.

### 3.3 Multi-factorial analysis

**Fig. 4:**
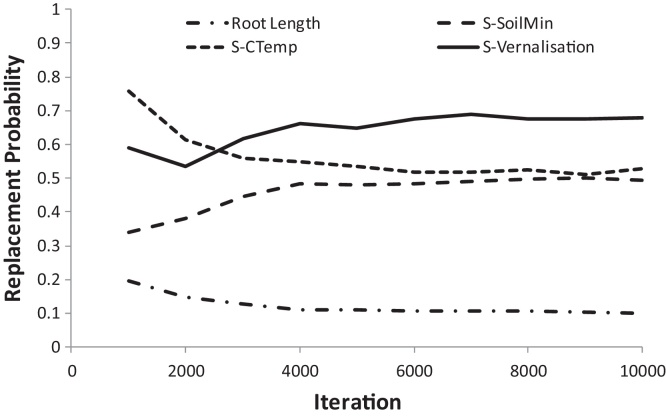
Evolution of the replacement probability for a selection of the candidate variables over the course of the analysis (variable symbols are defined in Table 3).

The evolution of the estimated replacement probability for selected variables is shown in Fig. 4 to illustrate the gradual convergence of the computational analysis.

**Fig. 5:**
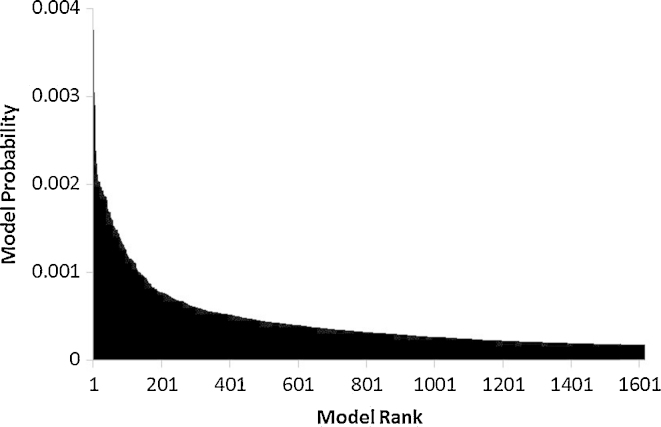
Model probabilities (*Qi* as calculated by Eq. (2)) in rank order for the models comprising 75% of the distribution (for presentation purposes model probability is calculated using the mean of the values derived from using *α* = 0.025, 0.05 and 0.1 in Eq. (2)).

The models comprising the uppermost 75% of the model probability distribution are shown in rank order in Fig. 5. This shows a small number of relatively better performing models, followed by a gradual decline in model performance. In comparison to previously published work using the replacement method (Crout et al., 2009; Tarsitano et al., 2011) the results here are notable for the large number of models with relatively similar performance.

The replacement probabilities are shown in Table 3. Values tending to unity imply that model performance improved when the variable was replaced (a noise variable). Values of 0.5 imply that model performance was unaffected when the variable was replaced by a constant (a redundant variable). Values tending to zero implied that model performance was worse when the variable was replaced.

The results in Table 3 were considered in the context of the mechanistic basis of the model design with a view to defining a minimum form of the Sirius model whose overall performance could be compared to the full model. Dry matter for grain filling was derived from a combination of photosynthesis during the grain filling period and translocation of stem and leaf biomass. The model recorded biomass at anthesis (BIOANTH) and this was used to define the rate at which stem and leaf biomass could be translocated to the filling grain. In effect translocation potential was related to biomass at anthesis. The reduction analysis suggested biomass at anthesis (BIOANTH) is redundant and that translocation potential could be a constant across sites and treatments. In the potential and drought treatments (where the data showed little difference in biomass at the time of anthesis) the effect of this replacement was to reduce the contribution of translocation to grain yield. In the case of *N* limited treatments, where anthesis biomass was relatively low, the effect was to increase the relative contribution of translocation to grain yield.

The model allowed for the expansion of the canopy to be reduced under water stress through the variable DrFACLAI and similarly the rate of canopy senescence increased through the variable GAKILR. These variables generally had low replacement probabilities implying that they contributed to the model's predictive skill.

The model used several interesting temperature adjustments to account for the differences between air temperature and soil minimum and maximum and canopy temperatures. Three model variables (ENAV, HCROP, TADJ) related to these adjustments were identified as redundant and as outlined earlier their overall importance was assessed through the inclusion of three switch variables (SwSOILMAX, SwSOILMIN, and SwCMAX) which had the effect of replacing each of these temperatures with the appropriate air temperature. All three were found to be redundant. Another interesting feature of Sirius is that the diurnal variation in temperature is used to estimate the rates of progress of a variety of plant processes rather than simply using the mean temperature. This feature was found to be redundant with replacement probabilities of approximately 0.5.

Sirius used plant nitrogen status to influence nitrogen uptake and to drive nitrogen translocation between the stem and leaf (Jamieson and Semenov, 2000). The variable MINSTEMDEMAND was calculated for each day and represented the minimum nitrogen demand which must be supported if growth was to occur. If this nitrogen was not available from uptake it was obtained through translocation of nitrogen from the leaf to the stem. MAXSTEMDEMAND provided an upper limit on crop nitrogen uptake in cases where stem nitrogen was high, causing nitrogen uptake to cease. Related to these variables LEAFDEMAND calculates the nitrogen required for the expected leaf expansion and used this to calculate appropriate transport of nitrogen between leaf and stem if that was required to sustain leaf expansion. MAXSTEMDEMAND was redundant in the analysis and MINSTEMDEMAND was borderline redundant. Given the values of the replacement constants the effect of these was to remove the influence of plant nitrogen status on nitrogen uptake, the crop simply removed whatever nitrogen was available to it. The replacement probabilities for LEAFDEMAND varied between the likelihood methods but overall were all <0.4. Therefore the minimum model simplified nitrogen uptake, ignoring the effect of plant nitrogen status on uptake, but retaining the use of leaf demand to drive internal nitrogen allocation. These variables do not relate to the link between crop nitrogen status and growth which remained unchanged in the minimum model.

Two variables associated with the prediction of nitrogen mineralisation were identified as redundant. These related to the influence of soil moisture (FQQ) and temperature (TA) on the rate of mineralisation. Both were replaced at the low end of their range with the effect of reducing nitrogen mineralisation to a low constant value.

The representation of crop development in Sirius combined the effect of temperature, including the effect of low temperatures (vernalisation), and daylength through simulation of the crop's leaf number. The approach is fully described by Jamieson et al. (1998a) and is only briefly summarised here. Leaves are produced at a constant thermal time interval (phyllochron) which is a cultivar specific parameter. During the course of the growing season the model sets a final leaf number depending on the vernalisation and daylength experienced by the growing crop according to the parameters defined for the cultivar. Anthesis occurred three phyllochrons after the point when the crop simulated leaf number is equal to the final leaf number. For cultivars with a vernalisation requirement a potential final leaf number was calculated in the vernalisation procedure. If the cultivar was daylength sensitive this potential final leaf number was further modified by a daylength function to set the final leaf number.

Two variables related to the vernalisation submodel (POTLFNO and PRIMORDNO) were found to be redundant, moreover the switch variable that turns off vernalisation entirely had a replacement values of *c*. 0.7 indicating that model predictions were improved when this variable is replaced.

Several variables related to soil surface evaporation (EVsoil) were redundant (ALPHA, CONDUC, PTSOIL, SLOSL) such that EVsoil is effectively replaced by a constant low value of 0.32 mm day^−1^. However ignoring soil surface evaporation completely (i.e. EVsoil = 0) had a detrimental effect on model performance, with the implication that although the model may be over-estimating evaporation, it did need to be considered. In the model soil evaporation influences both the calculation of soil maximum temperature and the soil water budget. Replacing soil evaporation with a constant continued to have an advantage for model performance even when soil maximum temperature was set equal to the maximum air temperature implying that the changes to water budget are beneficial to model performance.

In addition to the replacement probabilities for individual variables shown in Table 3 joint probabilities were calculated to indicate whether there were cases where the replacement of one variable was dependent on whether another variable was, or was not replaced. These results (not presented) showed no notable interactions for the redundant variables.

The analysis described is partial as the observational data used do not provide a test for all aspects of the model, nor do they represent a fully comprehensive range of site conditions. The interpretation of the results of the analysis needs to reflect these limitations if useful insights into the model design are to be gained. For example, although setting translocation potential to a constant improved model predictions in our analysis the replacement would be problematic if modelled anthesis biomass was lower than the proposed constant for setting the potential for translocation (9258 g m^−2^), as might be the case under extreme stress. This finding may imply that translocation potential is not linearly related to biomass and rather than simply set the variable to a constant it may be more productive to consider this feature of the crops behaviour more carefully in future model development.

Similar arguments apply to the redundancy of variables related to vernalisation. We are not suggesting that there is no such process as vernalisation, rather that the modelled modification of leaf number to account for vernalisation gives a worse result than that obtained by ignoring the process in the model. This may provide an indication that the representation of the process in the model is inappropriate. However caution is required, the findings were dictated by the response of the model to the range of conditions experienced over the 3 UK sites as New Zealand trials used a spring wheat cultivar.

Redundancy in the variables related to nitrogen mineralisation also illustrates the effect of partial data on the analysis. These variables had little effect on model performance as the crops nitrogen supply was high relative to the rate of nitrogen mineralisation. This is true even in the treatment with no nitrogen fertiliser additions where the nitrogen supply is dominated by the residual soil inorganic nitrogen at the time of planting.

### 3.4 Minimum model

On the basis of the above analysis a variant of Sirius was developed in which all the identified redundant model variables were reduced with the aim of comparing the resultant predictions with those of the full model. The differences were that (a) canopy and soil temperatures were taken as equal to the air temperature; (b) there was no allowance for diurnal variation in temperature on development or other processes; (c) nitrogen uptake is simplified such that the plant simply removes all nitrogen available to it in each time step; (d) the effect of vernalisation is ignored, with development being driven solely by temperature and photoperiod; (e) soil nitrogen mineralisation and soil surface evaporation were ignored; (f) translocation potential was considered a constant.

The resulting model predictions are compared to the full model in Fig. 1 and the summary Nash–Sutcliffe statistics are presented in Table 2. Notwithstanding these simplifications of the model the performance was almost identical to the full model, with slightly improved performance for leaf area and grain weights.

### 3.5 Conclusions

In previous applications of the variable replacement approach to model reduction all the models investigated were found to have redundant variables (Crout et al., 2009; Gibbons et al., 2010; Cox et al., 2006; Tarsitano et al., 2011). Similarly Sirius was found to contain variables whose use was redundant for predicting the data we have used in this analysis. However, most of the model's variables could not be reduced to a constant; of the 1 1 1 variables considered 16 were ultimately identified as potentially redundant.

The areas of the model where there was evidence of redundancy were (a) carbon translocation; (b) nitrogen physiology; (c) adjustment of air temperature for various modelled processes; (d) allowance for diurnal variation in temperature; (e) vernalisation (f) soil nitrogen mineralisation (g) soil surface evaporation. A minimum form of the model in which these features were either removed or replaced by constants performed slightly better than the full model with these data sets. This does not imply that these processes are not important in the real crop system. Rather, it indicates that the model's predictive performance was not improved through their representation in the model.

The outcomes of the work we have described depended on our choice of comparison data. In our case this was within season measurements of multiple components of the crop over a relatively small number of trials. We focussed on challenging the mechanisms within the model at a relatively detailed level in order to evaluate which of the modelled processes are contributing to the overall prediction of growth and development over the growing season. Therefore the approach is analogous to the type of detailed model inter-comparison described by Jamieson et al. (1998b). However our work could be described as a model intra*-*comparison as it was based on the comparison of many simplified forms of the same model. The approach provides automation to increase the efficiency of the evaluation and is a systematic means of increasing the rigour of the evaluation. However there is, as yet, no way to avoid the need for mechanistic model understanding and interpretation if model performance is to be critically evaluated.

The analysis is dependent on the observational data used. Subject to this limitation it provides a test of whether a particular formulation of model variables contributes to the models predictive performance. The aim should not be to simply find a simpler model and use it, but to use the identification of redundant variables as a means to challenge and improve the formulation used in the model.

## Figures and Tables

**Table 1:** Summary of the experimental trials used for the analysis.

Trial	Sites	Cultivar	Treatments	Year	Measurements made (figures in parenthesis are the number of observational data available)
NZ water stress (Jamieson and Semenov, 2000; Jamieson et al., 1998a)	Lincoln	Batten spring wheat	4 levels of rain shelter	1991–2	Time series of total biomass (48), grain biomass (35), LAI (59)
UK intensive management (Gillett et al., 1999)	Sutton Bonington, Boxworth, Gleadthorpe	Mercia	No treatments, maximum inputs	1992–3	Time series of total biomass (72), grain biomass, LAI (41), leaf number (74) and anthesis date (3)
UK nitrogen stress (Gillett et al., 1999)	Sutton Bonington	Mercia	Applied nitrogen levels of 0 and 90 kg ha^−1^	1992–3	Time series of total biomass (25), grain biomass (12), LAI (12)

**Table 2:** Nash–Sutcliffe (Nash and Sutcliffe, 1970) values for the full and minimum reduced models for the prediction of total biomass, grain weight and leaf area over all the observations considered. In this case Nash–Sutcliffe represents the proportion of the weighted variation accounted for by the model, in order to be consistent with the measures used to summarise model performance in the replacement analysis.

	Full model	Minimum model
Total biomass	0.971	0.972
Grain weight	0.845	0.868
Leaf area	−0.05	0.11

**Table 3:** Symbols and function of the model variables considered in the multi-factorial analysis together with the range of the variable in simulations of the full model and replacement probabilities calculated using three values of *α* (Eq. (2) and further described in the main text).

Model variable	Function	Full model range	Replacement constant	Replacement probability		
Temperature adjustments				*α* = 2.50%	*α* = 5%	*α* = 10%
S_CTEMP	Switch to set canopy temperature to air temperature	n/a	0	0.55	0.54	0.53
S_SOILMAX	Switch to set the estimate of maximum soil temperature to maximum air temperature. Maximum soil temperature is used in the early stages of growth to estimate the temperature controlling plant processes.	n/a	0	0.47	0.46	0.46
S_SOILMIN	Switch to set the estimate of minimum soil temperature to minimum air temperature. Minimum soil temperature is used in the early stages of growth to estimate the temperature controlling plant processes.	n/a	0	0.49	0.49	0.50
S_HTEMP	Switch to remove the diurnal temperature adjustments so that the model uses daily mean temperature.	n/a	0	0.52	0.53	0.54
ENAV	A soil heat physics calculation feeding into the calculation of HCROP and maximum soil temperature.	0.027–19.7	0.027	0.42	0.46	0.47
HCROP	Numerator in the correction applied to air temperature to estimate canopy temperature.	−3.62–7.67	0.76	0.48	0.46	0.47
CONDUC	Denominator in the correction applied to air temperature to estimate canopy temperature in the canopy temperature correction	0.014–0.115	0.022	0.45	0.50	0.50
TADJ	A temperature correction based on mean air temp which feeds into maximum soil temperature	0–6.13	0.25	0.57	0.53	0.52

Nitrogen uptake						
MAXSTEMDEMAND	Maximum daily stem nitrogen uptake, calculated from maximum stem *N* concentration	0–88.8	5.44	0.48	0.47	0.47
LEAFDEMAND	Daily leaf nitrogen demand calculated from leaf expansion and leaf nitrogen requirements.	−4.28–5.73	5.32	0.18	0.29	0.36
MINSTEMDEMAND	Difference between stem nitrogen concentration and the minimum stem nitrogen (i.e. the stem nitrogen deficit) on whole crop basis. Setting this variable to removes plant control on nitrogen uptake so that uptake is limited only by soil supply.	0–58.5	0	0.41	0.43	0.44

Grain filling						
BIOANTH	Biomass at anthesis; used to determine the maximum biomass available for translocation during grain filling	0–12602	9259	0.61	0.57	0.54
Nitrogen mineralisation						
FQ_Q	Factor representing the influence of soil moisture on nitrogen mineralisation	0–1	0.39	0.55	0.54	0.53
TA	7-day moving average air temperature; used to estimate the influence of temperature on nitrogen mineralisation	−2–19.8	5.58	0.49	0.48	0.48

Leaf expansion						
GAKILR	Factor used to represent the effect of drought on the canopy senescence	0–23.4	5.25	0.23	0.27	0.29
DRFACLAI	Factor used to represent the effect of drought on canopy expansion	−0.438–1	1.0	0.15	0.20	0.26

Vernalisation						
POTLFNO	Potential leaf number, used in the calculation of vernalisation effect on crop development	0–23.94	9.77	0.44	0.46	0.47
PRIMORDNO	Primordia number, used in the calculation of vernalisation effect on crop development	0–10.84	9.74	0.60	0.54	0.50
S_VERNALISATION	Switch to remove the influence of vernalisation on crop development	n/a	0	0.76	0.71	0.68

Soil surface evaporation						
ALPHA	Factor representing the effect of canopy shading on soil surface evaporation	1–1.35	1.34	0.54	0.52	0.51
PTSOIL	Intermediate variable used in the calculation of soil surface evaporation.	0.014–7.68	0.33	0.58	0.57	0.56
SLOSL	Intermediate variable used in the calculation of soil surface evaporation.	0–409.7	8.36	0.43	0.43	0.43

Penman						
EW	Intermediate variable used in the calculation of vapour pressure deficit, in turn used for the calculation of evaporation	4.46–28.8	14.879	0.39	0.40	0.42
HSLOP_tmean	Intermediate variable used in the calculation of Priestly–Taylor evaporation	0.34–1.69	0.66942	0.45	0.45	0.44
WND	Wind speed, used in the calculation of Penman evaporation (from Priestly–Taylor evaporation)	0–9.3	6.8452	0.21	0.26	0.31
